# A Novel Partial Discharge Localization Method in Substation Based on a Wireless UHF Sensor Array

**DOI:** 10.3390/s17081909

**Published:** 2017-08-18

**Authors:** Zhen Li, Lingen Luo, Nan Zhou, Gehao Sheng, Xiuchen Jiang

**Affiliations:** Department of Electrical Engineering, Shanghai Jiao Tong University, Shanghai 200240, China; lizhen10161830@163.com (Z.L.); zhounan@sjtu.edu.cn (N.Z.); shenghe@sjtu.edu.cn (G.S.); xcjiang@sjtu.edu.cn (X.J.)

**Keywords:** partial discharges, wireless UHF sensors, RSSI fingerprint, localization, compressed sensing, substations

## Abstract

Effective Partial Discharge (PD) localization can detect the insulation problems of the power equipment in a substation and improve the reliability of power systems. Typical Ultra-High Frequency (UHF) PD localization methods are mainly based on time difference information, which need a high sampling rate system. This paper proposes a novel PD localization method based on a received signal strength indicator (RSSI) fingerprint to quickly locate the power equipment with potential insulation defects. The proposed method consists of two stages. In the offline stage, the RSSI fingerprint data of the detection area is measured by a wireless UHF sensor array and processed by a clustering algorithm to reduce the PD interference and abnormal RSSI values. In the online stage, when PD happens, the RSSI fingerprint of PD is measured via the input of pattern recognition for PD localization. To achieve an accurate localization, the pattern recognition process is divided into two steps: a preliminary localization is implemented by cluster recognition to reduce the localization region, and the compressed sensing algorithm is used for accurate PD localization. A field test in a substation indicates that the mean localization error of the proposed method is 1.25 m, and 89.6% localization errors are less than 3 m.

## 1. Introduction

Partial Discharge (PD) is the representation of the insulation degradation of the power equipment in a substation, which can give rise to equipment failure and even serious accidents [1,2]. Therefore, PD detection and localization is an effective way to monitor the status of power equipment and avoid accidents. With the development of the smart grid, intelligent Condition Monitoring and Diagnostics (CMD) is one of the key requirement of a substation [3,4]. This paper proposes a novel partial discharge localization system in a substation that can quickly locate the power equipment with potential insulation defects.

**Figure 1:**
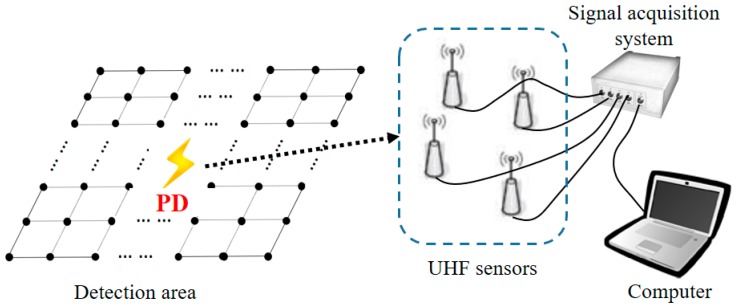
Traditional time difference of arrival (TDOA)–based Partial Discharge (PD) localization system in a substation.

PD can be detected by the measurement of ultrasonic waves, light, heat, and Ultra-High Frequency (UHF) electromagnetic waves [5]. For PD localization in a substation, the UHF method is one of the most effective ways due to its excellent stability, anti-interference, transmission distance, and transmission speed [6,7]. Typical UHF methods are mainly based on time or angle information of the UHF signals such as time of arrival (TOA) [8], time difference of arrival (TDOA) [9,10], and angle of arrival (AOA) [11,12]. Among them, TDOA has been widely used for PD localization. In this paper [13,14] we proposed a PD localization method in transformers based on TDOA. In the literature, [15] TDOA was used for PD localization in a substation. A traditional TDOA-based PD localization system in a substation is shown in Figure 1. When PD happens, the UHF sensors receive the UHF signals produced by PD. Then, by analyzing the time difference information, the location of PD can be estimated. Since the transmission speed of UHF signals is close to the speed of light, the time difference of the UHF signal’s arrival among UHF sensors is on a nanosecond level, which requires a high sampling frequency and high-precision time synchronization between the UHF sensors and the signal acquisition system. This requirement produces high hardware costs and makes TDOA-based localization hard to achieved.

**Figure 2:**
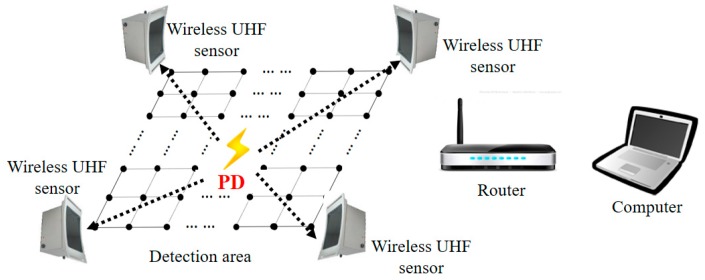
The received signal strength indicator (RSSI) fingerprint PD localization system in substation.

In recent years, the received signal strength indicator (RSSI) localization method has been widely studied in indoor localization, which uses only the signal strength information and is easily achieved by a low hardware cost solution [16,17]. In this paper [18], an RSSI-based PD localization systems is proposed, which uses the signal propagation model to estimate the distances and then calculates the location of PD. This method is easily implemented but is susceptible to the complex environment in substation. With the development of wireless UHF sensors, the arrangement of the UHF sensor array can be more flexible, which motivates us to use the RSSI fingerprint localization method for PD localization in a substation [19]. The RSSI fingerprint localization method is a scene analysis method with excellent environment adaptability and accuracy. The RSSI fingerprint PD localization system is shown in Figure 2. First, in the offline stage, a set of test points are arranged in the detection area. The RSSI values that are produced by discharging source, which denotes the received UHF signal strength, are measured at each test point. The RSSI values are transmitted from wireless UHF sensors to the computers though a router. All the RSSI values and corresponding test points’ coordinates are recorded to establish the RSSI fingerprint map of the detection area. Second, in the online stage, when PD happens, the RSSI values of PD are measured by the wireless UHF sensors array and used to estimate the location of PD by pattern recognition in the prebuilt RSSI fingerprint map. The purpose of our proposed method is to quickly find the faulty equipment in substations. Due to the intrinsic nature of the measuring process, our method cannot be applied to localize PD sources inside power equipment like transforms or gas-insulated substations.

Typical pattern recognition algorithms such as K Nearest Neighbor (KNN) [20] and neural networks [21] are easy to implement, but the performance in practical application is not satisfactory. In this paper, we use the compressed sensing (CS) algorithm to obtain more accurate PD localization. CS theory indicates that the sparse signal can be reconstructed by solving the *l*_1_-minimization problem [22,23]. For the proposed localization method, a localization result is a specific point in the detecting area. Therefore, the result vectors are sparse in nature. According to the CS theory, the accuracy of the CS localization algorithm decreases with the increase of test points [24]. Therefore, the online stage also consists of two steps. First, the preliminary localization is implemented by cluster recognition, which produces a small RSSI fingerprint map. Second, more accurate localization is obtained by CS algorithm with the use of a small RSSI fingerprint.

From practical application point of view, an affinity propagation clustering algorithm is designed and performed in the offline stage to eliminate the influence of interference signals and abnormal RSSI values for the fingerprint map. Furthermore, the built RSSI fingerprint map will also be divided into several clusters by this clustering algorithm for the preliminary localization in the online stage, as described above.

A field test is performed in a substation to verify the effectiveness of our proposed PD localization method. The results indicate that the proposed localization method shows satisfactory performance. The mean localization error is 1.25 m, and 89.6% of localization errors are less than 3 m.

The remainder of this paper is organized as following: Section 2 introduces the overview of the proposed PD localization method. The data acquisition and processing method and affinity propagation clustering algorithm in the offline stage are proposed in Section 3. Section 4 introduces the preliminary localization by cluster recognition and the accurate localization based on CS algorithm in the online stage. Section 5 introduces the hardware design. A field test and corresponding results are reported and analyzed in Section 6. Section 7 highlights the key contributions of this paper.

## 2. Overview of the Proposed PD Localization Method

**Figure 3:**
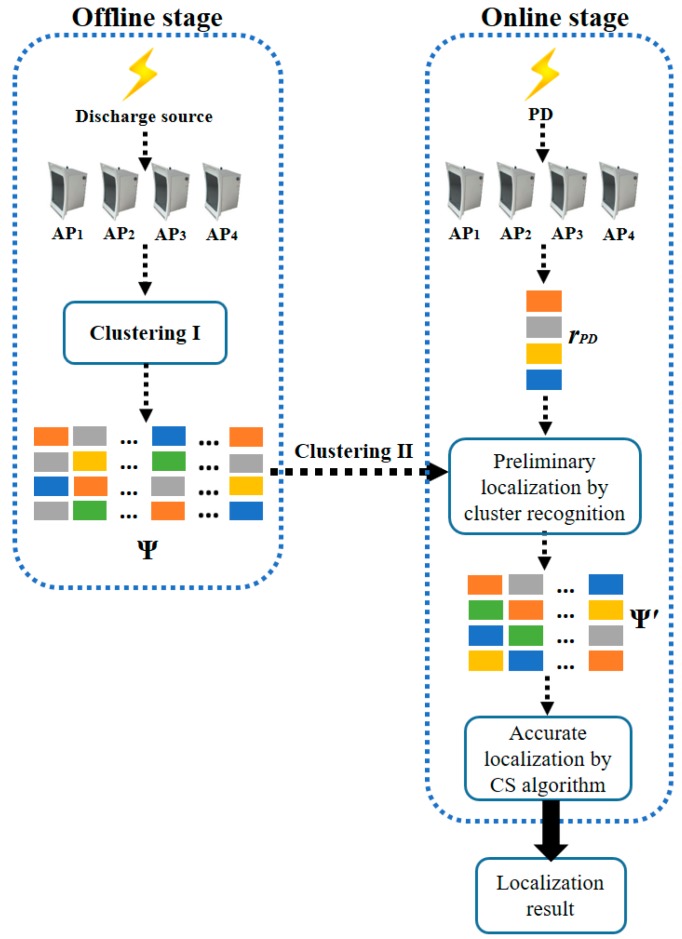
The flow chart of proposed PD localization algorithm.

The proposed RSSI fingerprint-based PD localization method is divided into two stages. Figure 3 shows the flow chart of the proposed PD localization method using four sensors, *AP*_1_, *AP*_2_, *AP*_3_, and *AP*_4_, as an example.

First, in the offline stage, totally *N* test points are arranged in the detection area, which are denoted by *RP_j_* (*j* = 1, 2, …, *N*). Then, the discharge source is used to produce PD at each test point, and the corresponding RSSI values are measured by *L* wireless UHF sensors. These wireless UHF sensors are denoted by *AP_i_* (*I* = 1, 2, …, *L*). Assuming the RSSI value of the test point *RP_j_* measured by the wireless UHF sensor *AP_i_* is denoted by *φ_i,j_*, then the RSSI fingerprint of *RP_j_* is,

(1) $$r_{j}={\left[\varphi_{1,j},\varphi_{2,j},\ldots ,\varphi_{L,j}\right]}^{T}$$

The RSSI fingerprints of all test points establish the RSSI fingerprint map Ψ of the detection area, which is denoted by,

(2) $$\Psi =\left(\begin{array}{cccc} \varphi_{1,1} & \varphi_{1,2} & \ldots & \varphi_{1,N}\\ \varphi_{2,1} & \varphi_{2,2} & \ldots & \varphi_{2,N}\\ \vdots & \vdots & \ddots & \vdots \\ \varphi_{L,1} & \varphi_{L,2} & \ldots & \varphi_{L,N} \end{array}\right)$$

There are two clustering processes in the offline stage. The Clustering I process is implemented during the establishment of the RSSI fingerprint map to reduce the influence of PD interference signals and abnormal RSSI values. The Clustering II process is implemented when the RSSI fingerprint map has been established. After the Clustering II process, the RSSI fingerprint map is divided into several clusters, which is the preparation for cluster recognition in the online stage.

Second, in the online stage, when PD happens, the RSSI fingerprint of PD is measured by wireless UHF sensor array and denoted as *r*_PD_,

(3) $$r_{PD}={\left[{\varphi^{\prime }}_{1},{\varphi^{\prime }}_{2},\ldots ,{\varphi^{\prime }}_{L}\right]}^{T}$$

where *φ*′*_i_* is the RSSI value of PD measured by *AP_i_*.

Then, pattern recognition is performed to find the column in Ψ that is most approximate to *r*_PD_. The coordinate of the matched column is the localization result. For better localization accuracy, the pattern recognition consists of two steps. In the first step, the preliminary localization is implemented by cluster recognition to get the small RSSI fingerprint map Ψ′. In the second step, more accurate localization is got by CS algorithm.

## 3. Offline Stage

### 3.1. Data Acquisition and Processing

In the offline stage, the RSSI fingerprint map of the detection area is established by site survey. During the site survey, a discharge source is used to produce PD at each test point, and the RSSI fingerprint data is acquired by wireless UHF sensors.

Since the strength of the discharge source is uncertain, the use of the RSSI fingerprint map constituted by the original RSSI values will produce conspicuous errors in pattern recognition. To solve this problem, the original RSSI values should be normalized.

Assuming the original RSSI fingerprint of *RP_j_* is,

(4) $${\hat{r}}_{j}={\left[{\hat{\varphi }}_{1,j},{\hat{\varphi }}_{2,j},\ldots ,{\hat{\varphi }}_{L,j}\right]}^{T}$$

where ${\hat{\varphi }}_{i,j}$ (*i* = 1, 2, …, *L*) are the original RSSI values of *RP_j_*. Then the normalization process is,

(5) $$\varphi_{k,j}=\frac{{\hat{\varphi }}_{k,j}}{\frac{1}{L}\sum_{t=1}^{L}{\hat{\varphi }}_{t,j}}$$

where *k* = 1, 2, …, *L*.

The normalization process highlights the spatial position relationship between the discharge source and sensors while decreases the influence of PD strength to improve the accuracy of pattern recognition.

### 3.2. Affinity Propagation Clustering

In the offline stage, two clustering procedures are performed as following.

#### 3.2.1. Clustering I

**Figure 4:**
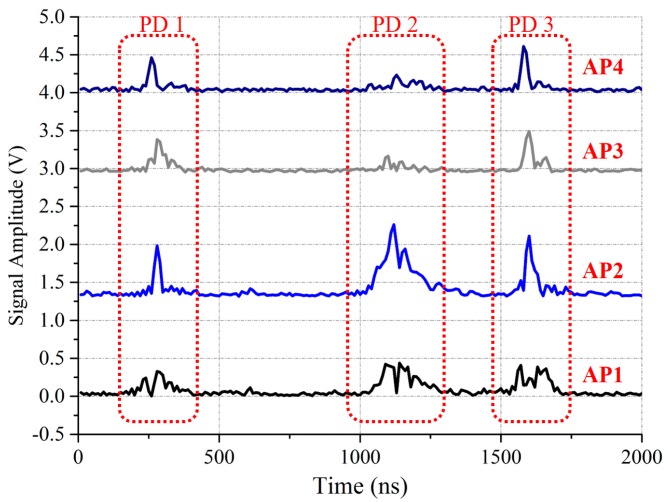
The digital envelope detection waveforms, which illustrate the PD interference phenomenon in the offline stage.

**Figure 5:**
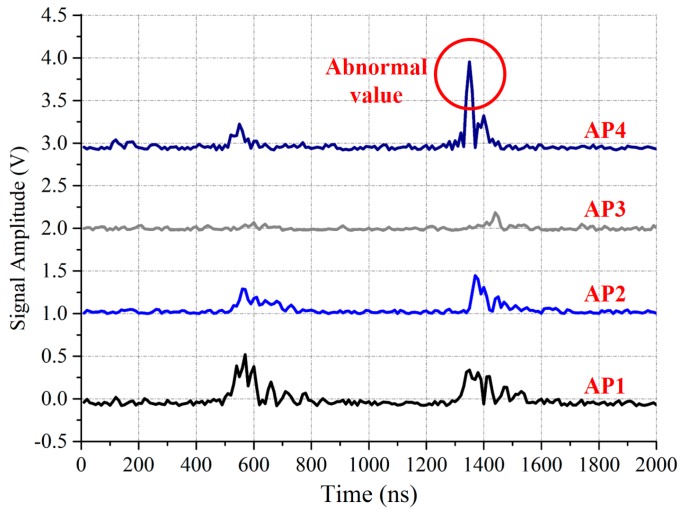
The digital envelope detection waveforms, which show the abnormal RSSI values phenomenon in the offline stage.

The object of Clustering I is to reduce the influence of PD interference in the offline stage as shown in Figure 4. During the measurement of RSSI fingerprint map, there are three PD signals detected by four wireless UHF sensors: *AP*_1_, *AP*_2_, *AP*_3_, and *AP*_4_ within about 1500 ns. It is hard to choose which PD is produced by our discharge source. Since the RSSI fingerprints measured in one test point are similar, we can eliminate the PD interference by following steps: first, producing PD at one test point for enough times and recording all the RSSI fingerprints. Then, clustering these fingerprints by the affinity propagation clustering algorithm. The mean value of the largest cluster is chosen as the RSSI fingerprint of this test point. Clustering I can also eliminate the abnormal RSSI values. As shown in Figure 5, these two PD signals are produced by one discharge source at one test point. The waveform received by *AP*_1_, *AP*_2_, and *AP*_3_ are similar, but the waveform received by *AP*_4_ contain conspicuous abnormal values. During the Clustering I process, these abnormal values will also be eliminated.

#### 3.2.2. Clustering II

The object of Clustering II is to divide the RSSI fingerprint map into several clusters for the preparation of cluster recognition in the online stage. The effect of Clustering II can be seen in Section 6.2.

The affinity propagation clustering algorithm is a high-efficiency clustering algorithm [25]. Therefore, it is chosen as the clustering algorithm for Clustering I and Clustering II. Affinity propagation clustering use the similarity matrix ***S*** to cluster the elements, denoted as,

(6) $$S=\left(\begin{array}{cccc} s(1,1) & s(1,2) & \ldots & s(1,D)\\ s(2,1) & s(2,2) & \ldots & s(2,D)\\ \vdots & \vdots & \ddots & \vdots \\ s(D,1) & s(D,2) & \ldots & s(D,D) \end{array}\right)$$

where *D* is the number of elements, and *s*(*i*, *j*) is the similarity between the element *i* and element *j*, which is denoted as,

(7) $$\left\{ \begin{array}{ll} s(i,j)=-{\left\|r_{i}-r_{j}\right\|}^{2} & \forall i,j\in (1,2,\ldots ,D),i\neq j\\ s(i,j)=w & \forall i,j\in (1,2,\ldots ,D),i=j \end{array}\right.$$

where *w* is the is the control parameter to adjust the number of clusters.

The core operation of affinity propagation clustering algorithm is the iteration process shown in (8) and (9),

(8) $$res^{\left(t+1\right)}(i,j)=s(i,j)-\underset{j^{\prime }\neq j}{\max}\left\{ava^{\left(t\right)}(i,j^{\prime })+s(i,j^{\prime })\right\}$$

(9) $$\left\{ \begin{array}{ll} ava^{\left(t+1\right)}(i,j)=\min\left\{\begin{array}{l} 0,res^{\left(t\right)}(j,j)+\\ \sum_{i^{\prime }\neq i,j}\max(0,res^{\left(t\right)}(i^{\prime },j)) \end{array}\right\} & ,i\neq j\\ ava^{\left(t+1\right)}(i,j)=\sum_{i^{\prime }\neq i,j}\max(0,res^{\left(t\right)}(i^{\prime },j)) & ,i=j \end{array}\right.$$

where *res*(*i*, *j*) is the responsibility value sent from element *i* to element *j*, which reflects how suitably the element *j* serves as the exemplar for element *i*, *ava*(*i*, *j*) is the availability value sent from element *j* to element *i*, which reflects how suitably the element *i* chooses element *j* as its exemplar, *t* is the number of iterations, *i*′ ∈ (1, 2, …, D), *i*ʹ ≠ *i* or *j*, *j*′ ∈ (1, 2, …, D), *j*′ ≠ *j*.

For all elements *k* (*k* = 1, 2. …, *D*), the condition of becoming an exemplar is,

(10) $$res^{\left(t+1\right)}(k,k)+ava^{\left(t+1\right)}(k,k)>0$$

Let *E* denote the set of exemplars, then for elements, its exemplar is the closest one in *E* under similarity measurement.

When *t* reaches the maximum number of iterations *t_max_* or the clustering results remain unchanged in consecutive *t_con_* iterations, the iteration is terminated.

To solve the convergence problem of iteration, we use the damping factor *λ* during the updating of *res* and *ava*, as shown in (11) and (12),

(11) $$res^{\left(t+1\right)}(i,j)=(1-\lambda )res^{\left(t+1\right)}(i,j)+\lambda res^{\left(t\right)}(i,j)$$

(12) $$ava^{\left(t+1\right)}(i,j)=(1-\lambda )ava^{\left(t+1\right)}(i,j)+\lambda ava^{\left(t\right)}(i,j)$$

where the range of *λ* goes from 0 to 1. The increase of *λ* will improve the global searching ability of the algorithm and produces better convergence performance but also slow down the convergence rate. Since the clustering processes described here are only implemented in the offline stage, the *λ* tends to be a big value.

## 4. Online Stage

According to the CS theory, if the number of sensors *L* is in the order of log (*N*), the localization result vector can be recovered from the RSSI fingerprint of PD at high probability [26,27]. However, *L* is hard to reach the order of log (*N*) in most cases. Therefore, the localization is divided into two steps. First, the preliminary localization is implemented by cluster recognition to determine which cluster the RSSI fingerprint of PD belongs to in the RSSI fingerprint map Ψ. The recognized cluster is regarded as the small RSSI fingerprint map Ψ’. Second, the accurate localization is implemented by CS algorithm with the use of Ψ′.

### 4.1. Preliminary Localization by Cluster Recognition

To reduce the influence of the boundary problem, we use all the elements in RSSI fingerprint map to implement the cluster recognition rather than only using the exemplars. The cluster recognition finds,

(13) $$\min{\left\|r_{PD}-{\bar{r}}_{g}\right\|}^{2}\;s.t\;g\in (1,2,\ldots ,C)$$

where C is the number of clusters, and ${\bar{r}}_{g}$ is the average value of the RSSI fingerprints in the *g*th cluster.

The RSSI fingerprints in the optimal cluster constitute a small RSSI fingerprint map Ψ′. The size of Ψ′ can be adjusted by the parameters in the affinity propagation clustering algorithm.

### 4.2. Accurate Localization by CS Algorithm

Assuming the number of the elements in Ψ′ is *N*′, therefore Ψ′ is a *N*′ × *L* matrix, which is a subset of Ψ. If a PD happens at the test point *RP_j_*, the location of PD can be denoted as,

(14) $$F={\left[0,\ldots ,0,1,0,\ldots ,0\right]}^{T}$$

*F* is a *N*′ × 1 vector with all elements are marked as zero, except the element that denotes the position of *RP_j_*, which is marked as one. Then we have,

(15) $$r_{j}=\Psi^{\prime }F$$

Multiplying both side of (15) by Φ.

(16) $$\Phi r_{j}=\Phi \Psi^{\prime }F$$

Let *Y* = Φ*r_j_*, then we have,

(17) $$Y=\Phi \Psi^{\prime }F$$

As mentioned above, *F* is a 1-sparse vector. According to the CS theory, (17) is a standard form of CS algorithm [22]. Φ is a *m* × *L* measuring matrix where *m* is the number of measurements and *m* < *N*′. Let Θ = ΦΨ′, which is a *m* × *N*′ sensing matrix.

Literature [28] indicates that *F* cannot be well recovered from the measurement *Y* unless Θ satisfies the Restricted Isometry Property (RIP) condition, which is denoted as,

(18) $$1-\varepsilon \leq \frac{{\left\|\Theta F\right\|}^{2}}{{\left\|F\right\|}^{2}}\leq 1+\varepsilon$$

where ε ∈ (0,1).

However, it is hard to use (18) to judge whether the RIP condition is satisfied. Literature [29,30,31] indicate that if the correlation between Φ and Ψ′ is weak, the RIP condition can be satisfied at high probability. Since the correlation between the Gaussian random matrix and other matrix is weak [32], the measuring matrix Φ is set as the Gaussian random matrix.

To satisfying the RIP condition better, we use SVD (Singular Value Decomposition) operation to transform sensing matrix Θ. denoted as,

(19) $$\Theta =U\Delta V^{T}$$

where *U* is a *m* × *m* orthogonal matrix, ***V*** is a *N*′ × *N*′ orthogonal matrix. Δ is denoted by (20) and (21),

(20) $$\Delta =[\begin{array}{cc} P & 0 \end{array}]$$

(21) $$\left\{ \begin{array}{l} P=diag(\delta_{1},\delta_{2},\ldots ,\delta_{m})\\ \delta_{1}\geq \delta_{2}\geq \ldots \geq \delta_{m} \end{array}\right.$$

Let

(22) $$\Delta^{*}=\left[\begin{array}{c} P^{*}\\ 0 \end{array}\right]$$

(23) $$\left\{ \begin{array}{l} P^{*}=diag(\frac{1}{\delta_{1}},\frac{1}{\delta_{2}},\ldots ,\frac{1}{\delta_{m}})\\ \delta_{1}\geq \delta_{2}\geq \ldots \geq \delta_{m} \end{array}\right.$$

Combining with (17), we have,

(24) $$\Delta^{*}U^{T}Y=\Delta^{*}U^{T}\Theta F$$

(25) $$P^{*}U^{T}Y=\left[\begin{array}{cc} I_{m} & 0 \end{array}\right]V^{T}F$$

Let

(26) $$Y^{\prime }=P^{*}U^{T}Y$$

(27) $$Q=\left[\begin{array}{cc} I_{m} & 0 \end{array}\right]V^{T}$$

We have,

(28) $$Y^{\prime }=QF$$

where *Y*′ is a transformed RSSI fingerprint of PD and *Q* is the transformed sensing matrix. From (27), we know that *Q* is a nearly orthogonal matrix. According to the literature [24], *Q* satisfies the RIP condition.

Since *Q* is a *m* × *N*′ matrix and *m* < *N*′, (28) cannot be solved directly. According to the CS theory, by solving *l*_1_-minimization model *F* can be well recovered from the measurement *Y*′ [32,33]. As shown in (29),

(29) $$F^{\prime }=\arg\min{\left\|F\right\|}_{1}\;s.t.\;Y^{\prime }=QF$$

where *F*′ is the recovered location vector.

We use the greedy algorithm to solve (29) due to its fast computation speed and small algorithm complexity [34]. Typical greedy algorithm includes the orthogonal matching pursuit (OMP) algorithm [35], the compressed sampling pursuit matching (CoSaMP) algorithm [36], subspace pursuit (SP) algorithm [37], and the generalized orthogonal matching pursuit (GOMP) algorithm [38]. We will use these four algorithms for comparison in experiment.

Since PD may not happen at a test point exactly, *F*′ is a vector with several nonzero elements rather than 1-sparse vector in theory. In this paper, the location of PD is decided as the coordinate of biggest element in *F*′.

## 5. Hardware Design

### 5.1. Wireless UHF Sensors

**Figure 6:**
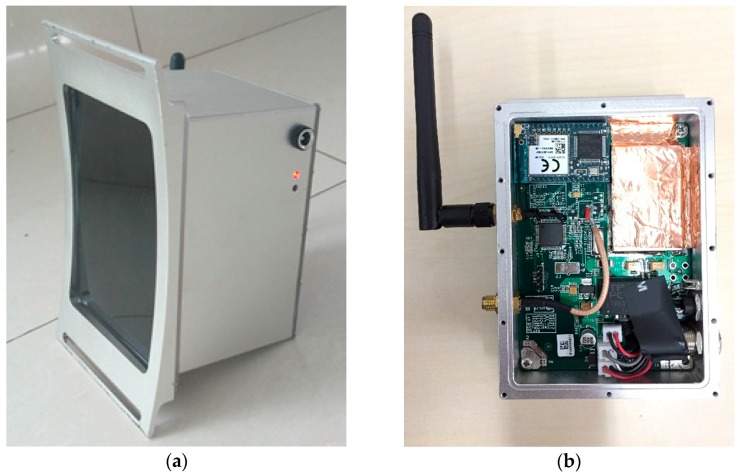
The picture of wireless Ultra-High Frequency (UHF) sensor. (**a**) Outside; (**b**) Inside.

The wireless UHF sensor used in the proposed PD localization method is shown in Figure 6. The antenna, bandpass filter, amplifier, detector, MCU, Wi-Fi module, and battery are concentrated in a metal shell. As shown in Figure 6b, the PD signal processing module (including filter, low noise amplifier, detector) is masked by copper foil for better anti-interference performance.

**Figure 7:**
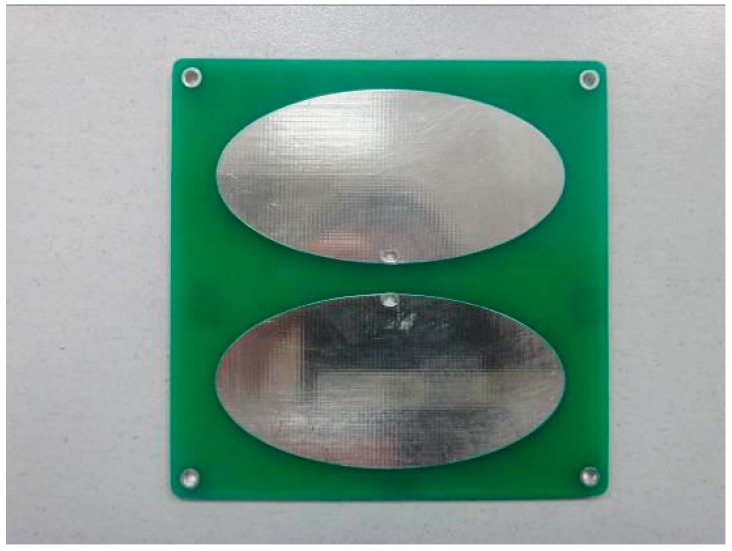
Elliptical dipole printed antenna panel.

**Figure 8:**
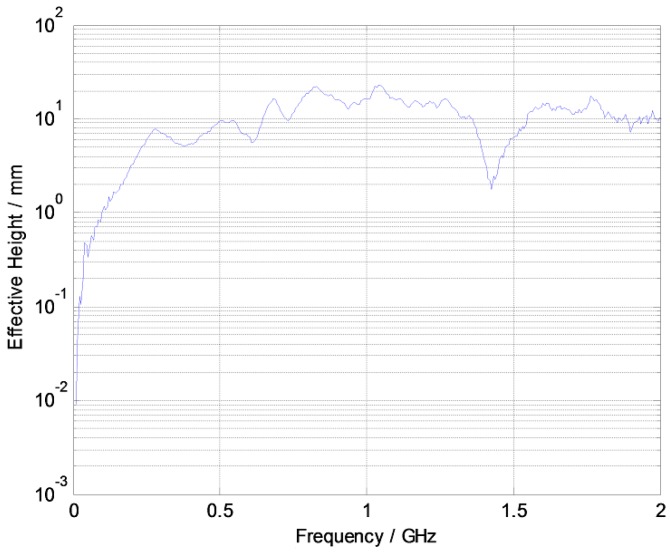
Sensitivity curve of the elliptical dipole printed antenna.

There are many kinds of antenna used in UHF sensors. Among them, the dipole antenna has the advantage of high sensitivity and small size. Therefore, we designed an PCB elliptical dipole antenna with a double feed and low cost. The panel of the elliptical dipole antenna is shown in Figure 7. The measured sensitivity curve is shown in Figure 8. The average effective height of our designed antenna is approximately 11.77 mm. The effective height denotes the ability of the antenna to transform the electromagnetic wave to voltage. Higher effective height will produce higher voltage.

**Figure 9:**
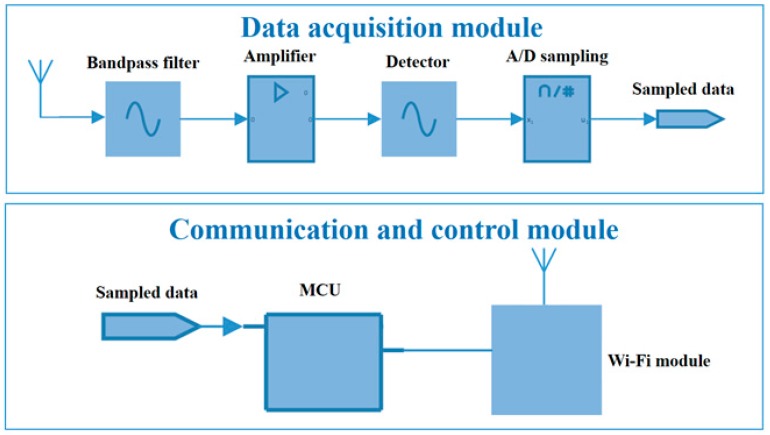
The diagram of wireless UHF sensor.

**Figure 10:**
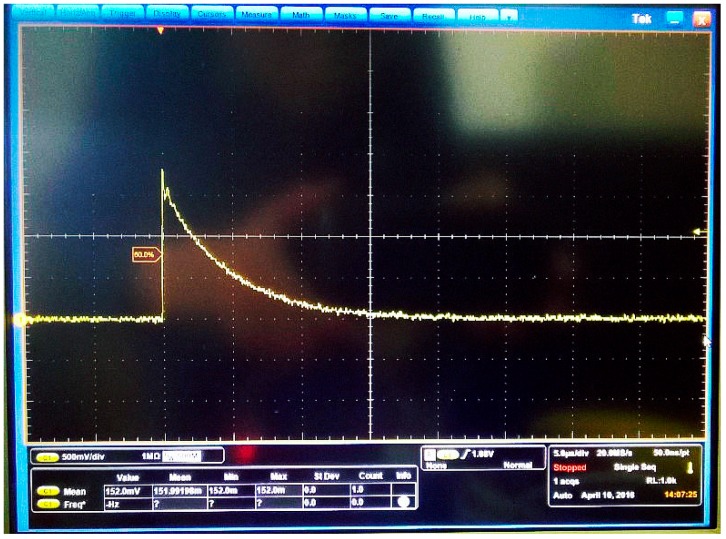
The detection waveform of a PD signal generated by PD emitter.

The process of the data acquisition by wireless UHF sensors is shown in Figure 9. First, the UHF electromagnetic wave is received by the antenna. Second, the detection waveform is obtained after signal conditioning by the bandpass filter, amplifier, and detector. Finally, via A/D sampling, the digital data are generated and transmitted to computer through a Wi-Fi module controlled by MCU. It is worthwhile to denoted that the sampling frequency is 2.7 MHz, which is much lower than that in TDOA because the goal of our method is to get the amplitude of waveforms rather than get the time difference information. The peak value of the detection waveform is the original RSSI value. A peak detector (AD8318) together with the peak holding circuit are designed to obtain the peak value of PD signals. Figure 10 shows the peak detection waveform of a PD signal generated by PD emitter. The waveform lasts about 15 us. Since the A/D sampling frequency is 2.7 MHz, there are about 40 sampling points, which can ensure that we obtain the peak value.

### 5.2. PD Source

**Figure 11:**
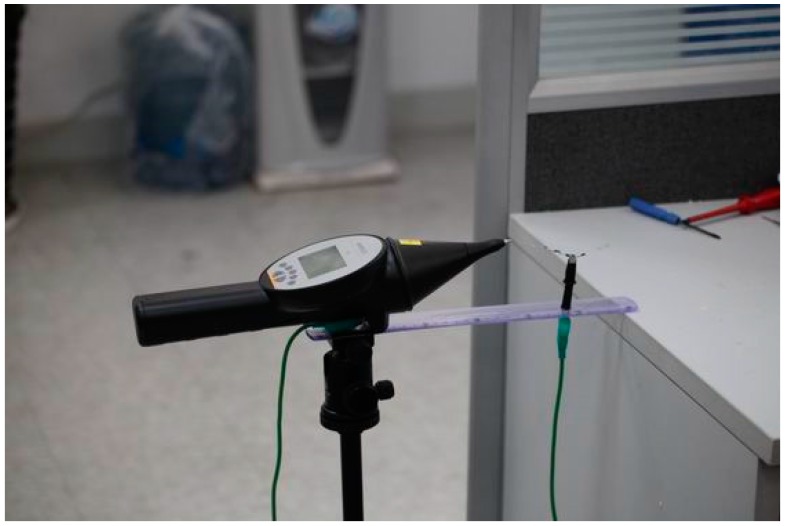
The PD simulator “EM TEST DITO”.

In the offline stage, the discharge source we used is a standard PD simulator named “EM TEST DITO” that can produce air discharge pulse according to EN/IEC 61000-4-2, as shown in Figure 11.

## 6. Experimental Verification

### 6.1. Experimental Scheme

**Figure 12:**
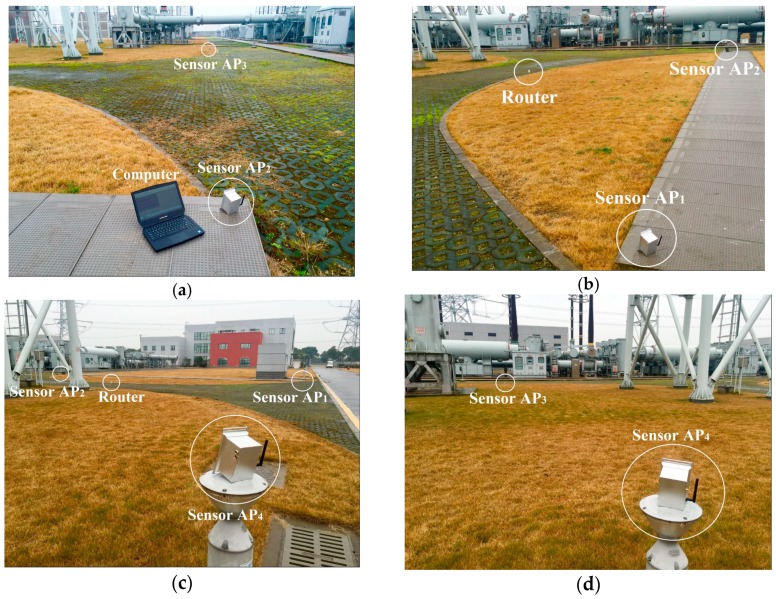
The picture of test site. (**a**) Picture 1; (**b**) Picture 2; (**c**) Picture 3; (**d**) Picture 4.

**Figure 13:**
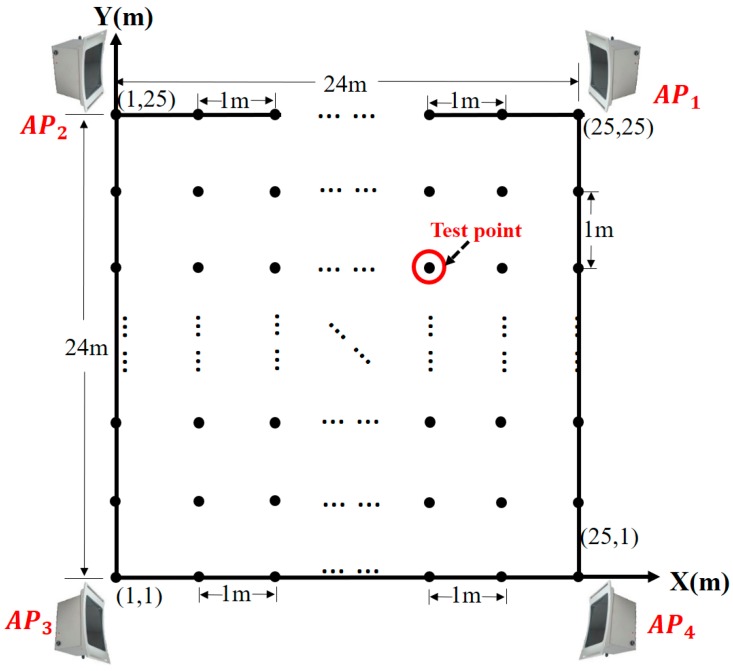
The plan of test site.

To verify the performance of proposed PD localization method, we chose a test site in a substation to perform a field experiment in a 24 × 24 m’ square area. Our PD localization system consists of a computer, a router, and four wireless UHF sensors, *AP*_1_, *AP*_2_, *AP*_3_, and *AP*_4_, as shown in Figure 12. From Figure 8 we can see that these wireless UHF sensors have a signal bandwidth of 300 MHz–1500 MHz and can transmit the envelope detection data of PD signal to a computer through Wi-Fi. To measure the RSSI fingerprint map of the test site, a total of 625 test points were arranged in the test site and distributed evenly with the adjacent distance of 1 m. The placement of sensors and test points is shown in Figure 13.

The test consists of two stages. First, in the offline stage, the RSSI fingerprint map Ψ was measured. A PD source was used to produce PD for 50 times at each test point. The RSSI fingerprint data was measured by wireless UHF sensor array and transmitted to a computer. By Clustering I process the RSSI fingerprint of each test point was obtained and these fingerprints constituted Ψ. Then the Clustering II process was implemented to divide Ψ into several clusters, which is the preparation for cluster recognition in the online stage. The parameters settings of the affinity propagation clustering algorithm are shown in Table 1.

Second, in the online stage, we used the standard discharge source to produce PD at one test point, and the RSSI fingerprint of PD was obtained. Then, the preliminary localization was implemented by cluster recognition, and the small RSSI fingerprint map Ψ′ was generated. Finally, the accurate localization was implemented by CS algorithm. This process was repeated at all the test points to verify the effectiveness of the proposed PD localization method.

### 6.2. Performance Evaluation of Offline Stage

**Figure 14:**
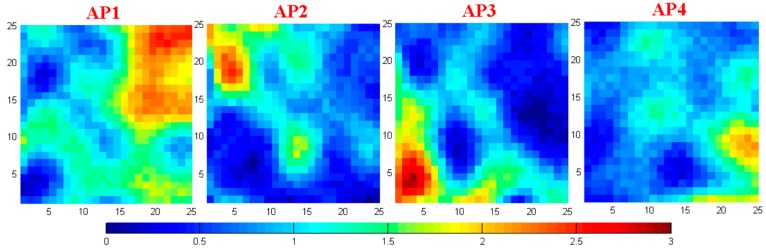
The RSSI fingerprint map measured by four sensors in the offline stage.

The RSSI fingerprint map Ψ of the test site measured in the offline stage is shown in Figure 14. Together with the plan of the test site in Figure 13, we find that the RSSI fingerprint map measured by each sensor is reasonable. The distribution of the RSSI values obeys the signal transmission properties and can reflect the spatial information of the test site. The irregular points in Ψ are caused by the influence of the complex electromagnetic and spatial environment in the substation.

**Figure 15:**
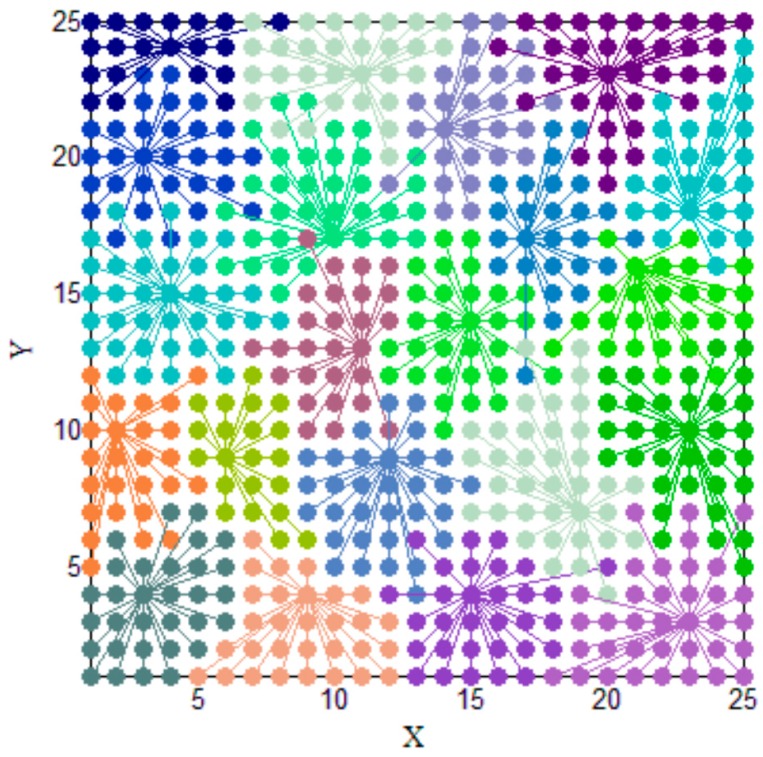
The clustering of Ψ.

By the Clustering II process, Ψ is divided into several clusters as shown in Figure 15. The RSSI fingerprints in Ψ are divided into 21 clusters. Therefore, the average number of fingerprints included in Ψ′ is about 30 (625/21). The performance of clustering is satisfactory because most of clusters are compact. This compactness indicates that if some test points are close to each other, their RSSI fingerprints are also similar. This property indicates that the distribution of RSSI fingerprints can reflect the spatial information of test site precisely.

In Figure 15, we can also find some elements in Ψ that are far from its exemplar. Most of this phenomenon appears at the boundary of clusters. Therefore, this phenomenon is mainly caused by measurement errors and the complex electromagnetic and spatial environment of the substation. Obviously, if PD happens at these test points, the localization error would be large.

### 6.3. Performance Evaluation of the Online Stage

First, for the preliminary localization, the performance of cluster recognition is satisfactory. About 81.9% of cluster recognition can recognize the correct cluster.

**Figure 16:**
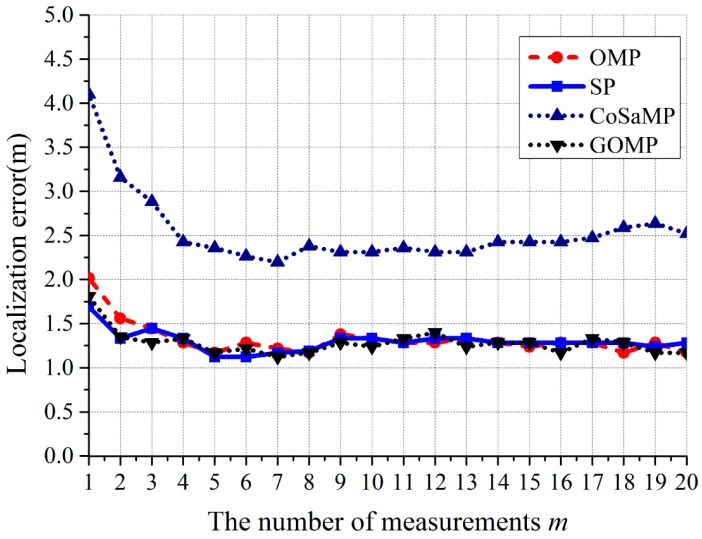
Relationship between localization error and the number of measurements *m* for OMP, CoSaMP, SP, and GOMP.

Second, for the CS algorithm, in order to choose the best solution for *l*_1_-minimization model, OMP, CoSaMP, SP, and GOMP were implemented for comparison. The results are shown in Figure 16. We can see that when *m* > 3, the localization errors are stable. The performance of OMP, SP, and GOMP are almost the same. Since the algorithm complexity of OMP is minimum, it is chosen as the solution for *l*_1_-minimization model finally.

**Figure 17:**
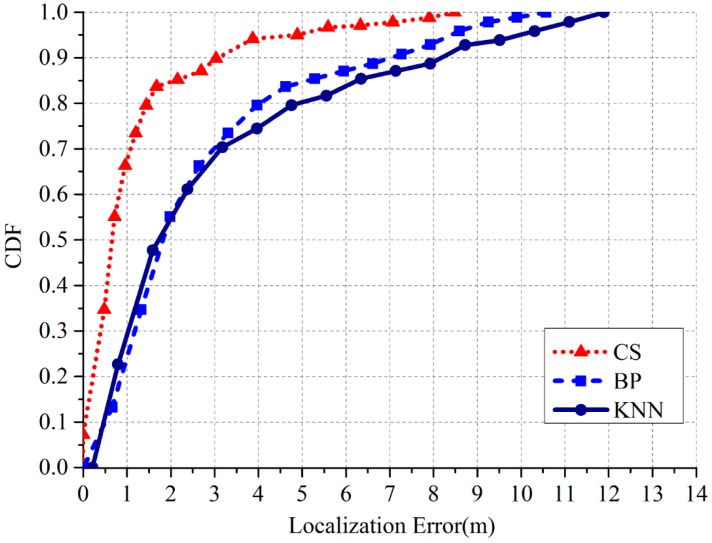
CDF of the localization errors under different pattern recognition algorithms.

Third, we compared three different pattern recognition algorithms: BP neural network, KNN, and the proposed CS algorithm. The localization results are shown in Table 2, and the corresponding cumulative distribution function (CDF) is shown in Figure 17. The results indicate that the performance of BP neural network and KNN is almost the same, while our proposed CS algorithm exhibits the best performance. The tendency of the CDF curve indicates that if the cluster recognition can recognize the correct cluster and generate a suitable Ψ′, the localization by CS algorithm would be accurate. We also investigated the execution times of these three algorithms. The average execution times of one PD localization is that: our CS algorithm needs 11.38 s; BP neural network needs 3.39 s and KNN needs 0.68 s.

**Figure 18:**
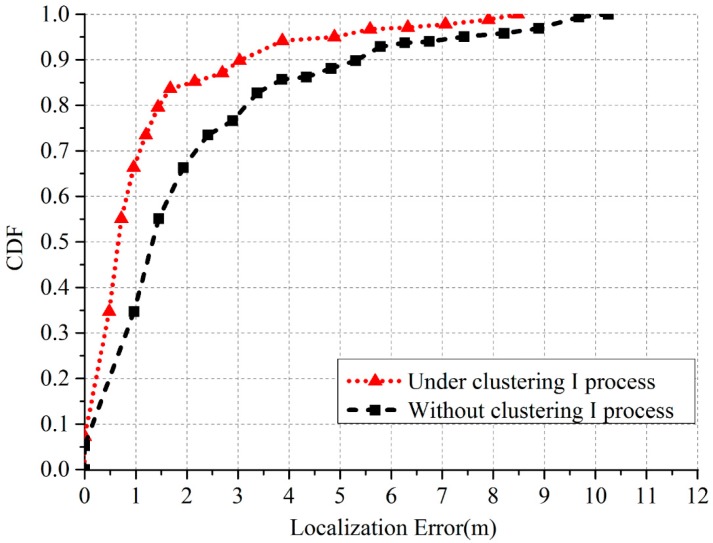
CDF of the localization errors under the Clustering I process and without the Clustering I process.

Finally, we investigated the performance of the Clustering I process. Table 3 gives the localization errors under the Clustering I process and without the Clustering I process. The corresponding CDF is shown in Figure 18. The Clustering I process can reduce 39.6% of the mean localization errors, which illustrates that the accuracy of RSSI fingerprint map is one of the key factors that determine the localization performance. The clustering I process will add extra execution times which rely on the actual situation of the site survey in the offline stage.

## 7. Conclusions

This paper proposes a novel PD localization method based on wireless UHF sensor array. The proposed PD localization method shows satisfactory accuracy through field test. Together with its low hardware cost, the proposed method is worthy to be widely applied in substations to find power equipment with potential insulation defects quickly. Considering the workload in the offline stage, the proposed method is more suitable in small and medium voltage level substations. In our future work, we plan to use the sub-regional monitoring strategy for larger substation and investigate the methodology to reduce the workload of fingerprint mapping. The weather conditions can also influence our proposed method. For example, in humid conditions, the PD interference will increase. Therefore, to guarantee the effectiveness of the clustering I process, the number of PDs produced by our discharge source in the offline stage should also be increased. However, according to the normalization process as addressed in Equation (5), the influence to the localization accuracy caused by weather conditions could be reduced to an acceptable level.

## Figures and Tables

**Table 1:** Parameters settings for affinity propagation clustering algorithm.

Parameters	*w*	*λ*	*t_max_*	*t_con_*
Values	−1.56	0.9	1000	100

**Table 2:** Localization results for different pattern recognition algorithm.

Parameters	CS	BP	KNN
Average error (m)	1.25	2.51	2.63
The proportion of the errors within 1 m	68.3%	24.2%	27.0%
The proportion of the errors within 3 m	89.6%	70.2%	68.1%
The proportion of the errors within 5 m	95.2%	85.0%	80.2%
The maximum error (m)	8.49	10.57	11.89

**Table 3:** Localization results under a different Clustering I process and without the Clustering I process.

Parameters	Under Clustering I Process	Without Clustering I Process
Average error (m)	1.25	2.07
The proportion of the errors within 1 m	68.3%	37.3%
The proportion of the errors within 3 m	89.6%	77.9%
The proportion of the errors within 5 m	95.2%	88.8%
The maximum error (m)	8.49	10.25

## References

[1] Portugues I.E., Moore P.J., Glover I.A., Johnstone C., McKosky R.H., Goff M.B., van der Zel L. (2009). RF-Based Partial Discharge Early Warning System for Air-Insulated Substations. IEEE Trans. Power Deliv..

[2] Fresno J.M., Ardila-Rey J.A., Martínez-Tarifa J.M., Robles G. (2017). Partial Discharges and Noise Separation using Spectral Power Ratios and Genetic Algorithms. IEEE Trans. Dielectr. Electr. Insul..

[3] Li C.R., Ma G.M., Qi B., Zhang G.J., Su Q. (2013). Condition monitoring and diagnosis of high-voltage equipment in china-recent progress. IEEE Electr. Insul. Mag..

[4] Arefifar S.A., Mohamed Y.A.R.I., EL-Fouly T.H. (2013). Comprehensive Operational Planning Framework for Self-Healing Control Actions in Smart Distribution Grids. IEEE Trans. Power Syst..

[5] Yaacob M.M., Alsaedi M.A., Rashed J.R., Dakhil A.M., Atyah S.F. (2014). Review on Partial Discharge Detection Techniques Related to High Voltage Power Equipment Using Different Sensors. Photon. Sens..

[6] Boya C., Rojas-Moreno M.V., Ruiz-Llata M., Robles G. (2015). Location of Partial Discharges Sources by Means of Blind Source Separation of UHF Signals. IEEE Trans. Dielectr. Electr. Insul..

[7] Ardila-Rey J.A., Martinez-Tarifa J.M., Robles G. (2015). Automatic Selection of Frequency Bands for the Power Ratios Separation Technique in Partial Discharge Measurements: Part II, PD Source Recognition and Applications. IEEE Trans. Dielectr. Electr. Insul..

[8] Hara S., Anzai D., Yabu T., Lee K., Derham T., Zemek R. (2013). A Perturbation Analysis on the Performance of TOA and TDOA Localization in Mixed LOS/NLOS Environments. IEEE Trans. Commun..

[9] Do J.Y., Rabinowitz M., Enge P. (2007). Robustness of TOA and TDOA Positioning Under Suboptimal Weighting Conditions. IEEE Trans. Aerosp. Electr. Syst..

[10] Hou H., Sheng G., Li S., Jiang X. (2015). A Novel Algorithm for Separating Multiple PD Sources in a Substation Based on Spectrum Reconstruction of UHF Signals. IEEE Trans. Power Deliv..

[11] Zhang R.B., Guo J.G., Chu F.H., Zhang Y.C. (2011). Environmental-adaptive indoor radio path loss model for wireless sensor networks localization. AEU-Int. J. Electron. Commun..

[12] Tomic S., Beko M., Dinis R. (2016). Distributed RSS-AoA Based Localization with Unknown Transmit Powers. IEEE Wirel. Commun. Lett..

[13] Robles G., Sánchez-Fernández M., Sánchez R.A., Rojas-Moreno M.V., Rajo-Iglesias E., Martínez-Tarifa J.M. (2013). Antenna Parametrization for the Detection of Partial Discharges. IEEE Trans. Instrum. Meas..

[14] Robles G., Fresno J.M., Martínez-Tarifa J.M. (2015). Separation of radio-frequency sources and localization of partial discharges in noisy environments. Sensors.

[15] Hou H., Sheng G., Jiang X. (2013). Robust Time Delay Estimation Method for Locating UHF Signals of Partial Discharge in Substation. IEEE Trans. Power Deliv..

[16] Chen W., Wang W., Li Q., Chang Q., Hou H. (2016). A Crowd-Sourcing Indoor Localization Algorithm via Optical Camera on a Smartphone Assisted by Wi-Fi Fingerprint RSSI. Sensors.

[17] Li B., Cui W., Wang B. (2015). A Robust Wireless Sensor Network Localization Algorithm in Mixed LOS/NLOS Scenario. Sensors.

[18] Zhang Y., Upton D., Jaber A., Ahmed H., Saeed B., Mather P., Lazaridis P., Mopty A., Tachtatzis C., Atkinson R. (2015). Radiometric Wireless Sensor Network Monitoring of Partial Discharge Sources in Electrical Substations. Int. J. Distrib. Sens. Netw..

[19] Iorkyase E.T., Tachtatzis C., Atkinson R.C., Glover I.A. Localisation of partial discharge sources using radio fingerprinting technique. Proceedings of the 2015 Loughborough Antennas Propagation Conference (LAPC).

[20] Bahl P., Padmanabhan V.N. RADAR: An In-Building RF-Based User Location and Tracking System. Proceedings of the IEEE INFOCOM.

[21] Youssef M., Agrawala A. (2008). The Horus Location Determination System. Wirel. Netw..

[22] Candes E.J., Wakin M.B. (2008). An Introduction to Compressive Sampling. IEEE Signal Process. Mag..

[23] Romberg J. (2008). Imaging via Compressive Sampling. IEEE Signal Process. Mag..

[24] Candes E.J., Tao T. (2006). Near Optimal Signal Recovery from Random Projections: Universal Encoding Strategies. IEEE Trans. Inf. Theory.

[25] Frey B.J., Dueck D. (2007). Clustering by Passing Messages between Data Points. Science.

[26] Zhang Y. (2013). Theory of Compressive Sensing via *l*_1_ Minimization: A Non-Rip Analysis and Extensions. J. Oper. Res. Soc. China.

[27] Candes E.J., Romberg J., Tao T. (2006). Stable Signal Recovery from Incomplete and Inaccurate Measurements. Commun. Pure Appl. Math..

[28] Candes E.J. (2008). The restricted isometry property and its implications for compressed sensing. Comptes Rendus Math..

[29] Baraniuk R.G. (2007). Compressive sensing. IEEE Signal Process. Mag..

[30] Baraniuk R.G., Davenport M.A., Devore R.A., Wakin M.B. (2008). A Simple Proof of the Restricted Isometry Property for Random Matrices. Constr. Approx..

[31] Candes E.J., Romberg J. (2007). Sparsity and Incoherence in Compressive Sampling. Inverse Probl..

[32] Tsaig Y., Donoho D. (2006). Extenstion of compressed sensing. Signal Process..

[33] Candes E.J., Wakin M.B., Boyd S. (2008). Enhancing Sparsity by Reweighted *l*_1_ Minimization. J. Fourier Anal. Appl..

[34] Ray A., Sanghavi S., Shakkottai S. (2015). Improved Greedy Algorithms for Learning Graphical Models. IEEE Trans. Inf. Theory.

[35] Tropp J.A., Gilbert A.C. (2007). Signal Recovery from Random Measurements via Orthogonal Matching Pursuit. IEEE Trans. Inf. Theory.

[36] Needell D., Tropp J.A. (2009). CoSaMP: Iterative signal recovery from incomplete and inaccurate samples. Appl. Comput. Harmonic Anal..

[37] Varadarajan B., Khudanpur S., Tran T.D. (2011). Stepwise Optimal Subspace Pursuit for Improving Sparse Recovery. IEEE Signal Process. Lett..

[38] Wang J., Kwon S., Shim B. (2012). Generalized Orthogonal Matching Pursuit. IEEE Trans. Signal Process..

